# Socioeconomic and environmental factors associated with malaria hotspots in the Nanoro demographic surveillance area, Burkina Faso

**DOI:** 10.1186/s12889-019-6565-z

**Published:** 2019-02-28

**Authors:** Toussaint Rouamba, Seydou Nakanabo-Diallo, Karim Derra, Eli Rouamba, Adama Kazienga, Yasuko Inoue, Ernest K. Ouédraogo, Moussa Waongo, Sokhna Dieng, Abdoulaye Guindo, Boukary Ouédraogo, Kankoé Lévi Sallah, Seydou Barro, Pascal Yaka, Fati Kirakoya-Samadoulougou, Halidou Tinto, Jean Gaudart

**Affiliations:** 1 Clinical Research Unit of Nanoro, Institute for Research in Health Sciences, National Center for Scientific and Technological Research, Nanoro, Burkina Faso; 2 Aix Marseille Univ, IRD, INSERM, UMR1252 Sciences Economiques & Sociales de la Santé & Traitement de l’Information Médicale, Marseille, France; 3 Center for Research in Epidemiology, Biostatistics and Clinical Research, School of Public Health, Université libre de Bruxelles (ULB), Brussels, Belgium; 4 Embassy of Japan in the Republic of Guinea, Conakry, Guinea; 5 Direction Générale de la Météorologie du Burkina Faso, Ouagadougou, Burkina Faso; 6 Ecole des Hautes Etudes en Santé Publique, Rennes, France; 7 MRTC, Malaria and Training Research Center – Ogobara Doumbo, Bamako, Mali; 8 Direction Régionale de la Santé du Centre-Ouest, Ministère de la santé, Koudougou, Burkina Faso; 9 Directorate of Health Information Systems, Ministry of Health, Ouagadougou, Burkina Faso; 10 Aix Marseille Univ, APHM, INSERM, IRD, SESSTIM, Hop Timone, BioSTIC, Marseille, France

**Keywords:** Malaria, Hotspots, Spatial epidemiology, Socioeconomic status, Meteorological factors, Spatio-temporal analysis, Bottleneck strategies, Lag time

## Abstract

**Background:**

With limited resources and spatio-temporal heterogeneity of malaria in developing countries, it is still difficult to assess the real impact of socioeconomic and environmental factors in order to set up targeted campaigns against malaria at an accurate scale. Our goal was to detect malaria hotspots in rural area and assess the extent to which household socioeconomic status and meteorological recordings may explain the occurrence and evolution of these hotspots.

**Methods:**

Data on malaria cases from 2010 to 2014 and on socioeconomic and meteorological factors were acquired from four health facilities within the Nanoro demographic surveillance area. Statistical cross correlation was used to quantify the temporal association between weekly malaria incidence and meteorological factors. Local spatial autocorrelation analysis was performed and restricted to each transmission period using Kulldorff’s elliptic spatial scan statistic. Univariate and multivariable analysis were used to assess the principal socioeconomic and meteorological determinants of malaria hotspots using a Generalized Estimating Equation (GEE) approach.

**Results:**

Rainfall and temperature were positively and significantly associated with malaria incidence, with a lag time of 9 and 14 weeks, respectively. Spatial analysis showed a spatial autocorrelation of malaria incidence and significant hotspots which was relatively stable throughout the study period. Furthermore, low socioeconomic status households were strongly associated with malaria hotspots (aOR = 1.21, 95% confidence interval: 1.03–1.40).

**Conclusion:**

These fine-scale findings highlight a relatively stable spatio-temporal pattern of malaria risk and indicate that social and environmental factors play an important role in malaria incidence. Integrating data on these factors into existing malaria struggle tools would help in the development of sustainable bottleneck strategies adapted to the local context for malaria control.

**Electronic supplementary material:**

The online version of this article (10.1186/s12889-019-6565-z) contains supplementary material, which is available to authorized users.

## Background

Malaria’s epidemiology is influenced by climatic factors [1–3] which affect the ecology of the vector and consequently exposure of human populations to pathogens. At a global or micro-epidemiological scale, malaria transmission is highly heterogeneous and modified by numerous factors, generating malaria hotspots that can maintain malaria transmission over a long time and across a wider area [4–7]. In 2015, according to the World Malaria Report, there were approximately 214 million cases of malaria and an estimated 438,000 deaths in malaria endemic countries, including Burkina Faso [7], with children under 5 years being the most affected [8].

In Burkina Faso, malaria is endemo-epidemic, the transmission is seasonal with a peak of incidence during and just after the rainy season and depends also on climatic and socioeconomic conditions [9]. Despite the combined efforts from local government and its international partners to mitigate the malaria burden, malaria annual incidence remains stubbornly high throughout the country areas. Additionally, the incidence of malaria increased from 309 cases per 1000 persons per year in 2011 to 514 cases per 1000 persons per year in 2016, however, the lethality due to malaria during the same period was considerably decreased (from 3.3% in 2011 to 0.9% in 2016 or 73% of reduction) [9, 10]. The current national policy is based on the Test-Treat-Track initiative (T3 initiative), universal distribution of long-lasting insecticide-treated nets (LLINs), seasonal malaria chemoprevention (SMC) for children under 5 years old during the high transmission period and intermittent preventive treatment (IPT) of malaria during pregnancy [11, 12]. Beside these measures, government adopted a national policy which provided health care free-of-charge to children under 5 years and to pregnant women attending public health facilities [13]. With these components of current national policy, it is noticeable that in 2017 and according to the national health statistics, malaria remained the first cause of outpatient consultations (43.5%), hospitalization (clinical observation) and (60.5%) mortality (35.9%) in health facilities; its annual incidence was estimated at 607 cases per 1000 persons per year with a lethality rate of 0.8% in the general population [14]. These statistics provide a partial estimate of the total malaria burden because home treatment or self-medication is a common practice in the Burkinabè context [15, 16], and are therefore not accounted for in the statistics presented above. To overcome the high rates of morbidity and mortality related to malaria, it is crucial to undertake research to refine approaches to applying existing interventions most effectively and efficiently in local contexts, in a bottleneck approach such as malaria hotspot-targeted strategies and according to the season of transmission [3]. Albeit some studies have reported that, within a micro-epidemiological scale in endemic areas, malaria disproportionately affects population living in similar conditions (nearest mosquito breeding site, wind direction and velocity, vegetation, house construction features, human genetic and behavioural factors) [17–21], the large growing studies carried out across African countries seemed to prove that malaria hotspot-targeted approaches are efficient and have more validity [22, 23]. However, for now the conclusion of results varied, some research have reported that malaria hotspot targeted approaches are not effective and/or efficient, especially for reducing transmission outside of the hotspot [4, 24].

Furthermore, albeit the effects of weather and environmental factors (social and natural) on malaria distributions at the global, regional and local scale (including the village level) are well documented [19, 25–30], controversial data regarding the role of meteorological and socioeconomic variables on generating or maintaining malaria hotspots observed at a fine scale remains a research topic to explore [17, 31–33].

In Burkina Faso, only few studies have directly or indirectly addressed spatial or spatio-temporal variation of malaria [5, 6, 34]. Some of these studies suggested relationships between malaria transmission and socioeconomic, environmental climatic variables [5, 6, 17, 18, 34, 35]. Nevertheless, until recently, the spatio-temporal dynamic of transmission at a fine geographical scale has not been sufficiently explored, because of lack of data. The literature investigating the role of socioeconomic and environmental factors on the dynamic spatio-temporal of malaria at the household level is growing. In such context of high malaria burden associated with national and local resource constraints in a framework of seasonal malaria chemoprevention program, we proposed to address this gap by analysing longitudinal malaria data from rural hyperendemic area in the Central-West region of Burkina Faso, Nanoro, taking into consideration socioeconomic and meteorological factors at household level.

The aim of this study was to define accurately the different transmission (or incidence) periods of malaria at a fine scale rural area and estimate the lag times between meteorological variables and high malaria incidence period. Then, to detect potential malaria spatial hotspot for each period of transmission. The study further investigated if meteorological and socioeconomic were associated to malaria hotspots observed at a fine scale over time.

## Methods

### Study area, design and population

**Fig. 1:**
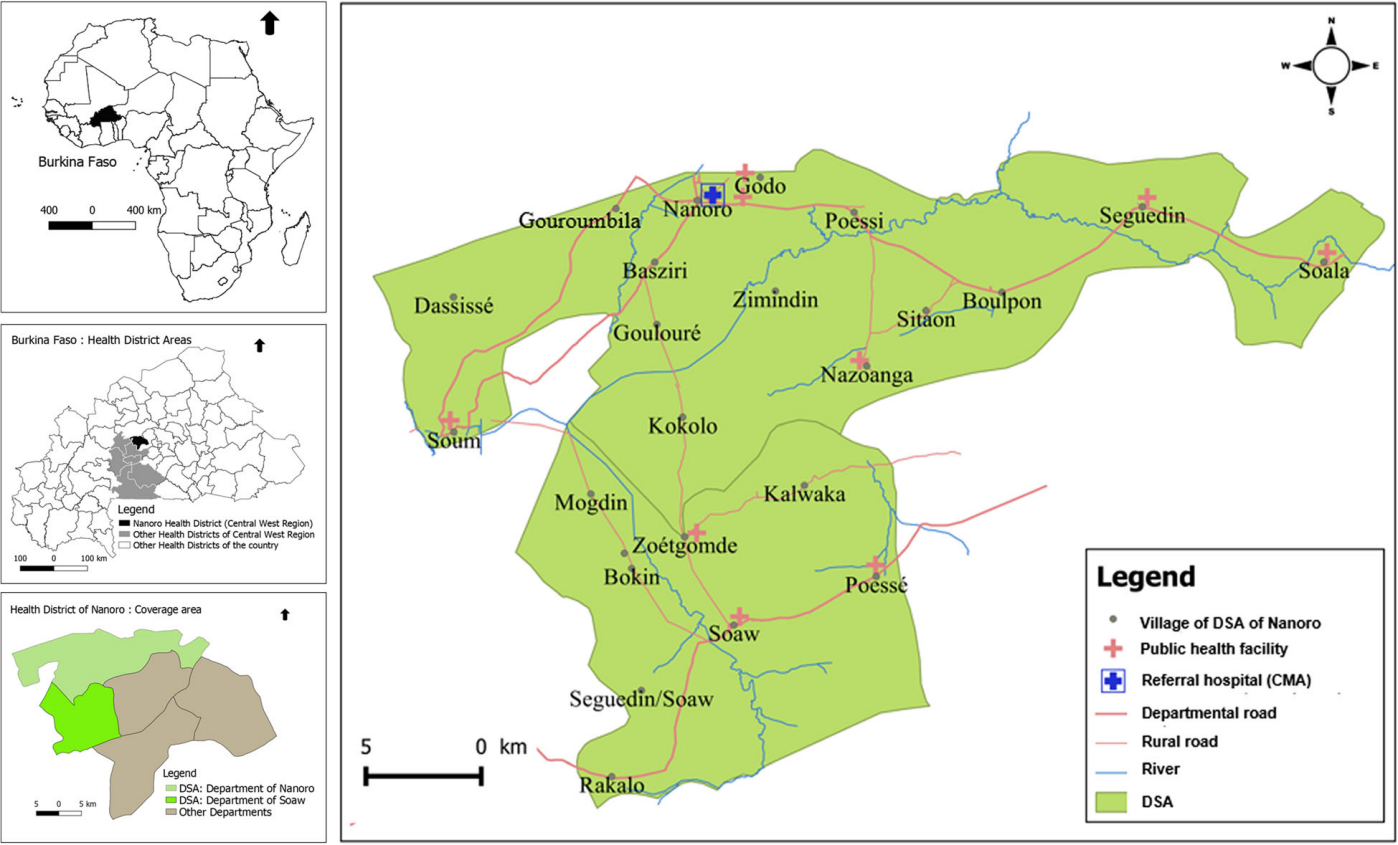
Burkina Faso map showing Nanoro Health District and the Nanoro Demographic Surveillance Area (DSA). Source: Burkina Faso, Base Nationale de Découpage du territoire (BNDT, 2006); shapefile downloaded from www.maplibrary.org. Created by Eli Rouamba, 2018

The study was carried out in Nanoro Demographic Surveillance Area (DSA), located in a rural in the Central West region of Burkina Faso. DSA was created in 2009 by the Clinical Research Unit of Nanoro (CRUN) and covered two departments: Nanoro (15 villages) and Soaw (9 villages). The DSA lied between longitudes 1°892,537 and 2°83,146 West and latitudes 12°857,955 and 12°872,863 North and covers an area of 594.3 km^2^. In this area, seven peripheral health facilities and one referral hospital (*Centre Médical avec Antenne Chirurgicale,* CMA) provided health care to the population. In the baseline of initial census (years 2009), 54,781 inhabitants were recorded. A unique identification number was assigned to each inhabitant of the DSA in order to track the different events occurring in the population by regular home visits [36]. This study focused on Nanoro departments health facilities (Fig. 1) [36, 37].

Our study was an observational, longitudinal cohort study. We examined all malaria cases reported in the telegram weekly official letter (*Telegramme Lettre Officiel Hebdomadaire,* TLOH) for 260 weeks (January 2010 to December 2014) from four health facilities of the Nanoro department (406.3 km^2^) covering 12 villages (35,952 inhabitants in 2010). The TLOH has been developed by the Burkina Faso National Epidemiological Surveillance department which provided weekly reports on 11 diseases (including malaria cases) notified in each health facility; the number of cases is then gathered and controlled by health districts each week before being sent to the Ministry of Health. All age groups patients attending health facilities within the DSA and for whom malaria diagnosis (according to national protocol) was confirmed by a parasitological exam (Rapid Diagnostic Test or Microscopy), were reported in the TLOH.

**Fig. 2:**
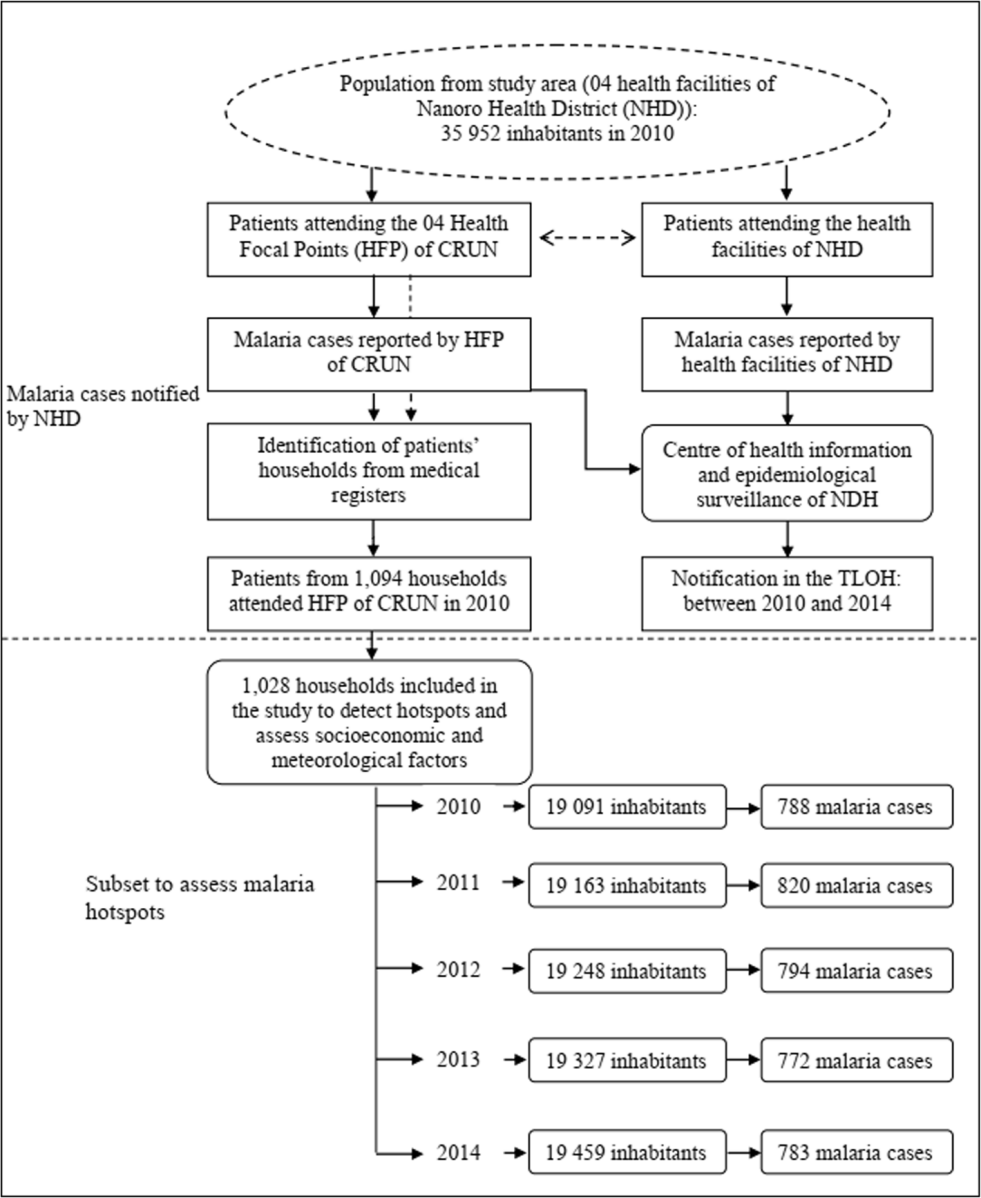
Flow chart of patients and their household’s selection

A subset from 1,028 households **(**Fig. 2) was further investigated, in which all individuals were included for detailed investigations, mainly for the detection of malaria hotspots and to assess the effect of socioeconomic and meteorological factors**.**

### Data acquisition procedures and data structure

#### Malaria cases data

Data on malaria cases were obtained from two sources. The first was extracted from TLOH of health facilities of Nanoro health district (NHD) and the second from medical consultation registries of health focal points (HFP) of CRUN. The CRUN’s HFP represented sentinel health stations that were set up in the framework of health research activities and nested within each health facilities of NHD [38].

The TLOH database included all malaria cases of the study area reported by health facilities (including case reported by HFP of CRUN). We extracted cases from four peripheral health facilities (Urbain, Godo, Nazoanga and Séguedin). This database aggregated malaria cases by health facility and by week (in accordance with the epidemiological calendar of the National Disease Control Directorate, Ministry of Health (*Direction de Lutte contre la Maladie* DLM). It was supplied by the Centre of Health Information and Epidemiological Surveillance (*Centre d’Information Sanitaire et de Surveillance Epidémiologique*, CISSE) of NHD.

Registries of HFP of CRUN: Each patient who was attending one of the HFP of CRUN was recorded in a registry by the medical team member who had examined him/her. Date of consultation, names, age and sex of the patient, village or neighbourhood he/she lived in, recent history of any treatment intake, weight, clinical signs, diagnosis, prescribed treatment (dose and duration) were reported.

#### Socioeconomic, demographic and geo-location data

This database included information from 1,028 households of individuals of HFP registries. All participants provided their consent to be part to a Health and Demographic Surveillance System (HDSS). Socioeconomic, demographic and Geo-location data were extracted from DSA database of CRUN. A unique identification number hold by each permanent resident [36] allowed us to establish the link between of individuals in the registries through a HDSS to their respective houses. Data on 26 variables of individual demography, household possessions and assets and materials for house construction were extracted. For this present study, analysis has been conducted at household level, so the individual data have not been considered.

Data on household included number of inhabitants per household, water source, house types and shapes, main goods and properties (for further precisions see [36]).

Geo-location data for each household was provided by GPS (Global Positioning System coordinates). These geo-location data were collected as part of the HDSS [36].

#### Meteorological data

These data were collected by the National Meteorological Directorate (*Direction Générale de la Metéorologie,* DGM), and aggregated weekly in accordance to the epidemiological calendar.

The data from local rainfall station located in Nanoro was used. Since temperature and humidity data were not available at departmental level, data from the synoptic station of Ouagadougou, located at about 85 km from the study area, were used, in accordance with the standards of the World Meteorological Organization (WMO). Thus, for evapotranspiration and temperature, WMO recommends a maximum distance of 150 km between the measuring stations. For the rainfall network, WMO recommends a minimum of one station per 10,000 km^2^ area [39]. The selected meteorological variables were rainfall (cumulative rainfall and number of rain events per week); temperatures (average of minimum and maximum per week, total average per week); relative humidity (minimum and maximum average per week, total average per week).

#### Geographical data

Nanoro department shape file (administrative boundary) with UTM zone 30 projection (Universal Transverse Mercator coordinate system zone 30) was downloaded from the following website: *www.maplibrary.org.*

### Statistical analysis

#### Descriptive and exploratory analysis of time series

Malaria incidence per week was estimated providing time series. Mann-Kendall test [40, 41] was used to assess the trend of the time series. The periodicities of the time series were assessed by their autocorrelograms, following the Box-Jenkins approach [42].

To take into account combinations of meteorological variables, but also to solve the collinearity and to reduce dimension, a principal component analysis (PCA) [43] was used, and the number of dimensions were selected according to the Kaiser rule*.*

To define transmission periods we performed, after a logarithmic transformation of the malaria incidence, a change point analysis [44], in order to detect significant changes in the mean and variance of the series for 260 weeks. For this purpose, the algorithm PELT *(*Pruned Exact Linear Time) [45] was used and the Modified Bayesian Information Criterion [46] was chosen for penalty.

#### Building ARIMA seasonal model and cross-correlation of the malaria series with the series of meteorological variables

Box-Jenkins approach [42] was used to model independently each time series. The best seasonal autoregressive integrated moving average (SARIMA) model was selected with the lowest Akaike Information Criterion (AIC). The remaining white noise was verified by using the Ljung-Box and Student test. With this approach, stationary time series were obtained, and used to explore relationships between time series.

Cross correlation function (CCF) was then used to assess the relationship between weekly meteorological variables (principal components), and log-transformed weekly malaria incidence.

#### Spatio-temporal analysis for hotspots detection

In our study a hotspot was defined using Martin Kulldorff’s Satscan approach [47] and was defined as statistical cluster or area of houses aggregation where malaria or incidence is higher than in the surrounding areas [4]. Spatial analysis for local hotspots detection was performed by using a purely elliptic spatial analysis (Poisson distribution), the detection was performed in each of the three combined period of malaria transmission (results of the change point analysis) in order to limit the impact of very high risk cluster on secondary cluster detection [48, 49]. The *p*-values were estimated by Monte Carlo inference.

Location of all household and statistically significant hotspots were mapped and roads, land, permanent water bodies were added using information from *OpenStreetMap,* after geo-referencing (https://www.openstreetmap.org/#map=12/12.6228/-2.1622).

#### Multivariable analysis

Based on Kulldorff scan detection results, the outcome variable was categorized as “being in a significant hotspot” (1) and “not being in a significant hotspot” (0) throughout the different transmission periods. Socioeconomic profile of each household and the effect of this profile on malaria hotspot were established by proceeding as follow. Firstly, we performed Multiple Correspondence Analysis (MCA) including all socioeconomic and demographic variables. The resulted coordinates (from the MCA) were then used for a hierarchical ascendant classification [50]. The end result grouped all the households in three clusters or “socioeconomic profiles”. Secondly, we used generalized estimating equations (GEE) model to explore the effect of socioeconomic profiles and principal components (by considering lag times between meteorological variables variable and high malaria transmission period) on malaria hotspot across time (malaria transmission periods). The accurate working correlation matrix was selected by assessing the correlation structure showing the low QIC (Quasi-AIC) score [51].

### Ethics consideration

This analysis used household socioeconomic data from Nanoro HDSS that set up a population-based monitoring system in a framework of main study entitle ‘Pharmacovigilance for ACTs in Africa’ and was approved by Centre Muraz Institutional Ethical Committee (N° Réf. 03-2010C/E-CM), Burkina Faso National Ethics Committee for Research in Health (N° 2010–27).

### Software and packages

Statistical analyses were performed using the R software version 3.2.5 (R Development Core Team, R Foundation for Statistical Computing, Vienna, Austria), including the following packages: *autoarima, Dcluster, FactoMineR, geepack, rgdal*. Local hotspot assessment was performed by using the SatScan™ software version 9.4.2. Maps were provided by using the QGIS software (2014 QGIS Development Team).

## Results

### Description of time series and subset characteristics

Over the 5 years, 115,306 malaria cases were notified in the TLOH. The annual malaria incidence per 1000 inhabitants were 559, 581, 613, 646 and 623 respectively for year 2010, 2011, 2012, 2013 and 2014. For the subset, 19,091 subjects from 1,028 households were included in 2010, among them 788 malaria cases were reported. The flow of the study subject selection is shown on Fig. 2.

**Fig. 3:**
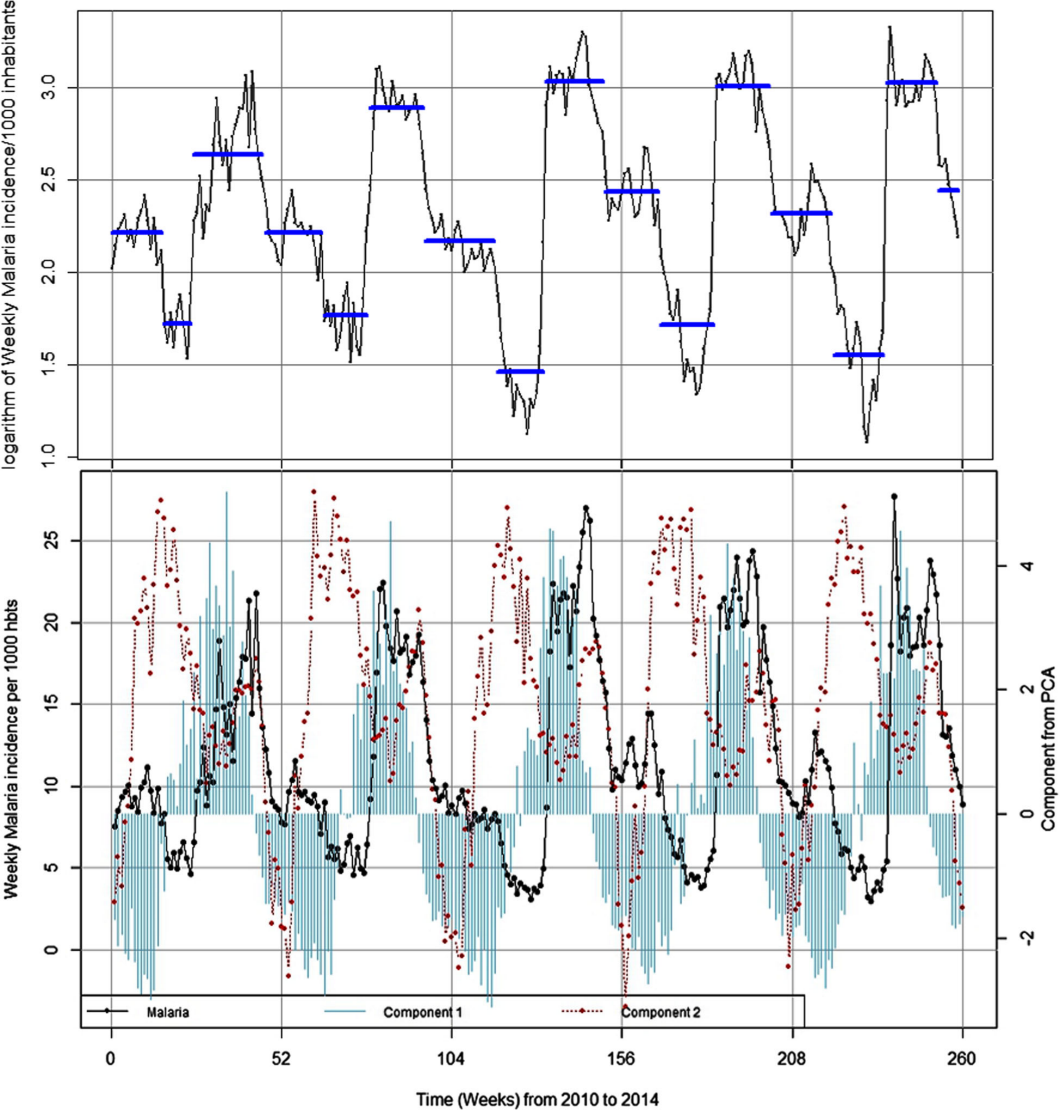
Transmission periods and seasonality of weekly malaria incidence and weekly meteorological variables from 2010 to 2014. Component 1 was associated to rainfall and relative humidity (cumulative rainfall and number of rain events and relative humidity); Component 2 was associated to temperatures (maximum, minimum and average)

The Fig. 3 which illustrates the evolution of malaria incidence and meteorological variables time series, showed a maximum peak every 52 week, indicating the classical seasonal pattern of this endemo-epidemic area, but no trend was observed (see Additional file 1).

The reduction of meteorological variables showed that the two first components explained 88.25% of the total inertia (see Additional file 2). The first component was mainly associated to rainfall (cumulative rainfall and number of rain events) and relative humidity (maximum, minimum and average). The second component was mainly associated to temperatures (maximum, minimum and average).

The cross-correlation analysis between malaria and the first component showed that rainfall and humidity were positively and significantly associated with malaria incidences with a time lag of 9 weeks. The lag time of 9 weeks indicates the time elapsing periods between the peak of rainfalls and the peak of malaria incidence (rise of malaria case). The second component showed that temperatures were positively and significantly associated with malaria cases with a time lag of 14 weeks. The time lagged variables were used in the GEE regression for components 1 and 2 in order to assess the impact of these meteorological factors on spatial hotspot genesis.

The change point analysis of the malaria time series identified 15 time-points or periods. These periods were uniformly distributed annually (three periods each year). The most important changes in the time series for malaria incidence for one period (i.e. 52 weeks) occurred between July and mid-November where malaria incidences increased (about 3.53-fold) (Table 1 and Fig. 3).

**Table 1:** Description of malaria incidence and rainfall according to the transmission periods

Year	Seasons	Start date	Seasons Duration^a^	Incidence^b^	Rainfall^c^	Rainfall with lag^d^
2010	Intermediate	2010-01-04	16	9.18	0.82	10.38
	Low	2010-04-26	9	5.64	16.99	37.32
	high	2010-06-28	22	14.40	28.13	12.86
	Intermediate	2010-11-29	18	9.20	0	6.18
2011	Low	2011-04-04	14	5.95	18.36	34.61
	high	2011-07-11	17	18.08	21.99	2.06
	Intermediate	2011-11-07	22	8.78	0	5.69
2012	Low	2012-04-09	15	4.49	19.69	47.05
	high	2012-07-23	18	20.91	31.22	1.47
	Intermediate	2012-11-25	17	11.50	0	6.30
2013	Low	2013-03-25	17	5.76	15.03	29.96
	high	2013-07-22	17	20.32	25.69	4.64
	Intermediate	2013-11-18	19	10.24	0.16	5.81
2014	Low	2014-03-31	16	4.88	19.18	32.36
	high	2014-07-21	16	20.64	23.7	3.62
	Intermediate	2014-11-10	7	11.63	0	0

^a^Seasons Duration in weeks

^b^Malaria incidence per 1000 person-weeks for the transmission season

^c^Accumulates Rainfall (mm) / week for the same transmission season

^d^Accumulates Rainfall (mm) / week with time lag (9 weeks)

### Spatio-temporal hotspot detection

**Fig. 4:**
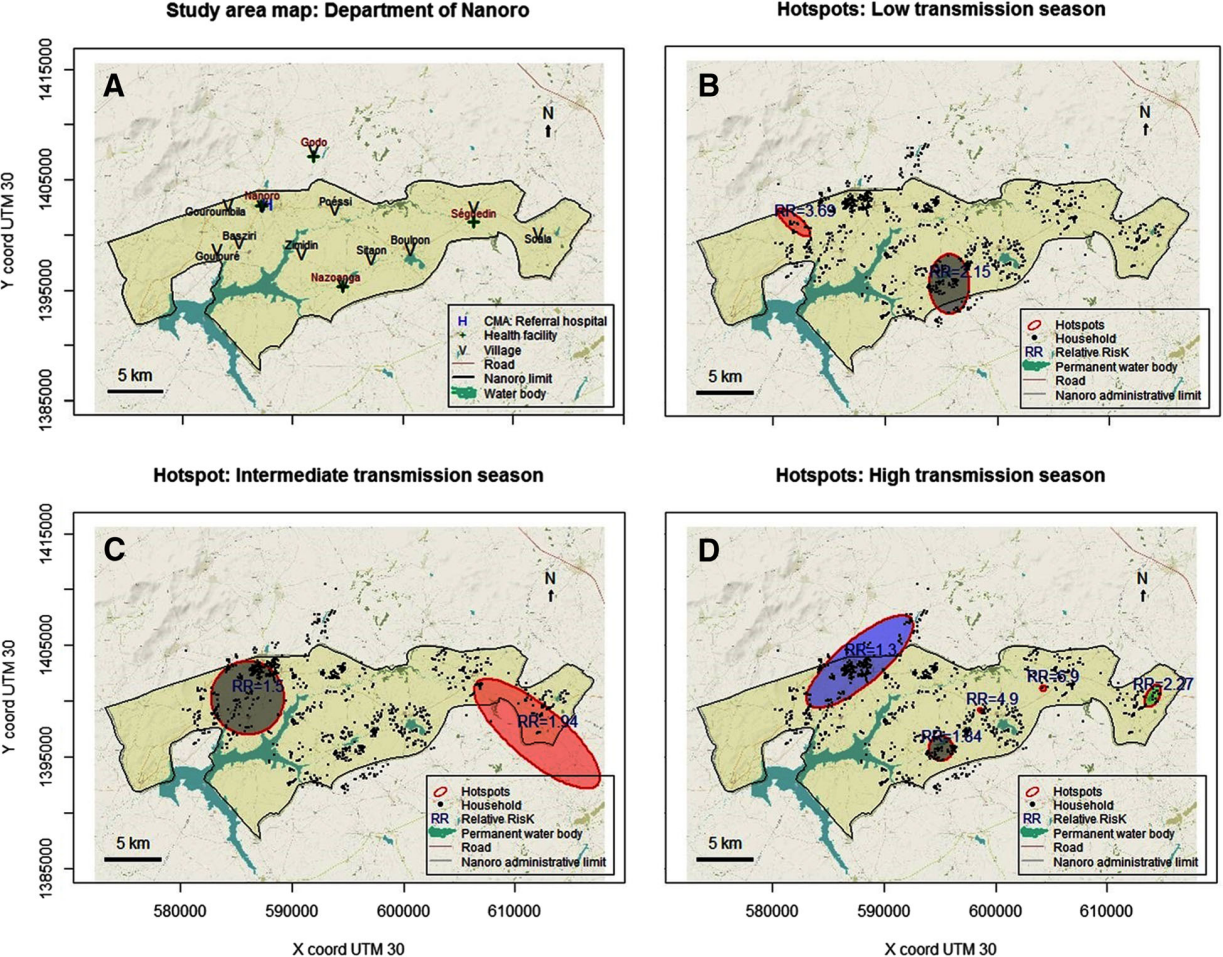
Map of Nanoro (**a**) with water bodies, villages and health facilities. Hotspots of cumulative weekly malaria incidence in Nanoro: High transmission period (**b**), Intermediate transmission period (**c**), Low transmission period (**d**). Source: Burkina Faso, Base Nationale de Découpage du territoire (BNDT, 2006); shapefile downloaded from www.maplibrary.org. The map background (raster) is captured from https://www.openstreetmap.org/#map=12/12.6228/-2.1622. Maps created by Toussaint Rouamba, 2018

Kulldorff scan method for hotspots detection, according to the three transmission periods, showed a spatio-temporal heterogeneity. But, the location of the different hotspots through the study area was relatively stable through the study period. Two significant hotspots were detected during the low and intermediate transmission periods, with relative risks (RR) of 2.15 and 3.69 (Low transmission period, respectively 82 and 13 households, *p* < 0.001), and of 1.50 and 1.94 (Intermediate transmission period, respectively 211 and 43 households, *p* = 0.001 and *p* = 0.028). Five significant hotspots were identified during the high transmission period. Principally, this area belonged to the villages of Gouroumbila, Nanoro, Basziri, Goulouré and Godo. The hotspot which showing the highest RR was in Séguedin village (RR = 6.90, 1 single household, *p* = 0.002). The largest hotspot counted 255 households, with the lowest RR of 1.30 (*p* = 0.011) and was located both in Nanoro and Godo Villages (Table 2, Fig. 4, and Additional file 3).

**Table 2:** Malaria hotspots detected by the elliptic scan

Period	N^a^	X ^*b*^	Y ^*b*^	Axis in km (major/minor)	Number of households	RR ^*c*^	*P* value
High^*H*^	1	595,099	1,395,750	1.09/1.09	38	1.84	< 0.001
	2	604,229	1,401,150	0.00/0.00	1	6.90	0.002
	3	614,102	1,400,450	1.12/0.56	12	2.27	0.003
	4	587,828	1,403,520	6.01/2.00	255	1.30	0.011
	5	598,740	1,399,110	0.31/0.15	2	4.90	0.015
Inter^*I*^	1	586,028	1,400,300	3.28/3.28	211	1.50	0.001
	2	611,969	1,397,070	7.09/2.36	43	1.94	0.028
Low^*L*^	1	595,715	1,395,570	2.67/1.78	82	2.15	< 0.001
	2	581,892	1,401,070	1.82/0.61	13	3.69	< 0.001

*L* Low transmission periods

*I* intermediate transmission periods

*H* high transmission periods

^a^Number of hotspots for each period

^b^Centroid coordinates of hotspots (UTM zone 30)

^**c**^*RR* (Relative risk)

### Multivariable analysis

In this study area, we find three socioeconomic profiles among the 1,028 households. Thus, 797 (77.5%), 219 (21.3%) and 12 (1.2%) households were classified (from HCA) as low, middle and high socioeconomic status respectively (Table 3). The low socioeconomic class was characterized mainly by a shorter distance to health facility (37.8%), less material goods ownership (excluding mobile phone, 89.7%, radio, 69.6%), less latrines (2.8%), piped water, no electricity and no gas, more houses made by clay bricks, dirty floors, and clay, wood, or straw made roofs.

**Table 3:** Socioeconomic characteristics of households (1028) obtained by the hierarchical ascendant classification

	Socioeconomic status of households			
	Low *N* = 797	Middle *N* = 219	High *N* = 12	Total *N* = 1028
Distance to health facility, n (%)				
< 5 km	540 (67.8)	127 (58.0)	5 (41.7)	672 (65.4)
5–10 km	251 (31.5)	87 (39.7)	7 (58.3)	345 (33.6)
> 10 km	6 (0.8)	5 (2.3)	0 (0)	11 (1.1)
Ownership of radio, n (%)				
No	242 (30.4)	38 (17.4)	4 (33.3)	284 (27.6)
Yes	555 (69.6)	181 (82.6)	8 (66.7)	744 (72.4)
Ownership of TV, n (%)				
No	788 (98.9)	159 (72.6)	0 (0)	947 (92.1)
Yes	9 (1.1)	60 (27.4)	12 (100)	81 (7.9)
Ownership of mobile phone, n (%)				
No	82 (10.3)	4 (1.8)	0 (0)	86 (8.4)
Yes	715 (89.7)	215 (98.2)	12 (100)	942 (91.6)
Ownership of fridge, n (%)				
No	797 (100)	206 (94.1)	0 (0)	1003 (97.6)
Yes	0 (0)	13 (5.9)	12 (100)	25 (2.4)
Ownership of car, n (%)				
No	796 (99.9)	199 (90.9)	8 (66.7)	1003 (97.6)
Yes	1 (0.1)	20 (9.1)	4 (33.3)	25 (2.4)
Ownership of motorcycle, n (%)				
No	322 (40.4)	47 (21.5)	0 (0)	369 (35.9)
Yes	475 (59.6)	172 (78.5)	12 (100)	659 (64.1)
Ownership of bicycle, n (%)				
No	14 (1.8)	10 (4.6)	1 (8.3)	25 (2.4)
Yes	783 (98.2)	209 (95.4)	11 (91.7)	1003 (97.6)
Toilet ownership, n (%)				
Latrine	3 (0.4)	66 (30.1)	11 (91.7)	80 (7.8)
Latrines unenriched	19 (2.4)	151 (68.9)	1 (8.3)	171 (16.6)
Absence	775 (97.2)	2 (0.9)	0 (0)	777 (75.6)
Major source of drinking water, n (%)				
Tap (Piped water)	2 (0.3)	6 (2.7)	7 (58.3)	15 (1.5)
Well	131 (16.4)	1 (0.5)	0 (0)	132 (12.8)
Water drilling	660 (82.8)	212 (96.8)	1 (8.3)	873 (84.9)
Other	4 (0.5)	0 (0)	4 (33.3)	8 (0.8)
Main source of lighting, n (%)				
Electricity	0 (0)	89 (40.6)	12 (100)	101 (9.8)
Other	797 (100)	130 (59.4)	0 (0)	927 (90.2)
Main material of walls (bedrooms), n (%)				
Made of cement bricks	7 (0.9)	59 (26.9)	11 (91.7)	77 (7.5)
Semi-hard	81 (10.2)	12 (5.5)	1 (8.3)	94 (9.1)
Made of clay bricks	709 (89.0)	148 (67.6)	0 (0)	857 (83.4)
Main material of the floor, n (%)				
Tiles	0 (0)	0 (0)	4 (33.3)	4 (0.4)
Cover floor with roughcast (cement)	568 (71.3)	216 (98.6)	8 (66.7)	792 (77.0)
Dirt floor	229 (28.7)	3 (1.4)	0 (0)	232 (22.6)
Main material of the roof, n (%)				
With iron sheets	720 (90.3)	219 (100)	12 (100)	951 (92.5)
Made of clay and wood	64 (8.0)	0 (0)	0 (0)	64 (6.2)
Made of straw and wood	13 (1.6)	0 (0)	0 (0)	13 (1.3)
Gas for cooking, n (%)				
No	797 (100)	216 (98.6)	4 (33.3)	1017 (98.9)
Yes	0 (0)	3 (1.4)	8 (66.7)	11 (1.1)
Electricity, n (%)				
No	797 (100)	130 (59.4)	0 (0)	927 (90.2)
Yes	0 (0)	89 (40.6)	12 (100)	101 (9.8)

Unadjusted univariate GEE analysis (Table 4) revealed significant associations between malaria hotspots and households classified as low socioeconomic status (OR = 1.23, 95% CI: 1.05–1.44 and the temperature component (OR = 0.65, 95% CI: 0.61–0.69). After adjusting on temperature and rainfall/humidity, households showing a low socioeconomic status presented the highest significant risk (aOR = 1.21, 95% CI: 1.03–1.40) associated to malaria hotspot compared to the households belonging to the medium and high status.

**Table 4:** Factors associated with malaria hotspots

	Univariate		Multivariable	
	OR [95% CI]	*P* value	aOR [95% CI]	*P* value
Socioeconomic status				
Medium (Ref)	1	–	1	–
Low	1.23 [1.05–1.44]	0.013^b^	1.21 [1.03–1.40]	0.021^b^
High	0.90 [0.43–1.92]	0.79	0.93 [0.43–1.98]	0.84
Component 1	1.03 [1.00–1.06]	0.05^**a**^	1.01 [0.97–1.05]	0.68
Component 2	0.65 [0.61–0.69]	< 0.001^c^	0.65 [0.61–0.69]	< 0.001^c^

Component 1: resumed rainfalls considering its lag time with malaria

Component 2: resumed temperatures considering its lag time with malaria

*aOR* adjusted odds ratio

^a^significant at the 0.1 level

^b^ significant at the 0.05 level

^c^significant at the 0.01 level

## Discussion

This longitudinal observational study showed the annual seasonal pattern of malaria incidence, but with an intermediate transmission (or incidence) period between the two-classical low and high transmission. Moreover, weekly rainfall was positively associated with weekly malaria incidence, with a lag time of 9 weeks. Our findings supported a relative stability of the spatio-temporal pattern. The relative stable hotspots were associated with meteorological factors but also with low socioeconomic status.

Contrary to literature that describes two malaria transmission periods (high and low) in Burkina Faso [17, 37], our results have shown three clear transmission (or incidence) periods per year in Nanoro setting which correspond to high, intermediate and low period of malaria incidence. Similar result was recently found by *Ouedraogo* et al. (2018) in Ouagadougou. Our intermediate period of malaria incidence (middle-November to March) is consistent with the rainfall transition period reported in the literature (November and February) [37]. During our “intermediate” period, recorded rainfalls were quasi null (Table 1), however, the presence of wetlands (temporary and permanent waterbodies) associated with optimum range of temperatures (28.41 °C [18°–38°]), relative humidity (30.33% [12–47%]), and human activities (off-season agriculture is intensifying during this period) created suitable conditions to maintain larval or mosquito abundance and thus contributed to maintain risk for malaria transmission in human populations.

Furthermore, considering the lag time (9 weeks) found in our study, rainfalls had a delayed influence on malaria cases whatever the transmission period (Table 1). These findings established the classical positive strong temporal association between meteorological factors (component 1: rainfall & relative humidity; and component 2: temperatures) and weekly malaria incidence in our area. In addition, these lag times coincided with the theoretical vector-parasite-host cycle under optimum conditions [52–55] and contributes to better understanding the classical hypothesis of biological/ecological drivers of the spatial-temporal distribution of malaria throughout a country. Indeed, surface water from first rainfalls may have been rapidly dried, by infiltration or evaporation. Therefore, the formation of a temporary waterbodies needed numerous rainfalls before becoming breeding sites. Another delay may be due to the vector life cycle itself, from eggs to adults, and the number of cycles before reaching the sufficient population needed to accelerating the parasite transmission (also depending on meteorological factors favouring mosquito survival). Finally, another delay may be observed until the first clinical cases were reported, defining the epidemic “official” onset.

In Ghana [56], neighbouring country of Burkina, in Ethiopia (East Africa) and China [1, 57–59], rainfalls and malaria were positively correlated with a lag time of 9 and 10 weeks respectively. Similarly, lag times between one and 3 months were reported in Mali (3 months), Kenya (one and 3 months) and China (1 month) [58, 60, 61]. By contrast, malaria incidence rate was delayed by 2 weeks compared to meteorological factors in Ouagadougou, located at about 85 km from Nanoro site. According to the authors of this latter study, the 5 dams located in this central region may contribute to the constant presence of vectors, which explains this short delay [6]. Taken together, this finding highlights the variability of spatio-temporal dynamic of malaria at micro-epidemiological scale in endemic areas. These lag times should be understood and considered by the Health Program Planners when implementing SMC campaigns in local context for delivering interventions at the right/relevant time.

However, studies carried out in Sri Lanka and Madhya Pradesh (Central India), did not detect a clear relationship between rainfall and malaria incidence probably because of dry areas [62] or flooded areas [63].

Our study area was characterised by a spatial aggregation and spatio-temporal heterogeneity of malaria cases through all transmission periods. Similarly, in Burkina Faso, a study that use the Kulldorff’s approach with health facility as spatial scale, had found also a spatial variability and relative temporal stability of malaria incidence around the capital Ouagadougou [6].

The persistence of hotspots, especially in the village of Nanoro (down-town of the department) and its surrounding, could be explained partly by several combined factors. First, by presence of several areas of off-season agriculture, better health services accessibility that could improve malaria cases reporting, the construction of the new dam of Soum which created a swampy area, favourable conditions for the breeding sites. Second, by the high population density which was estimated at 104 persons per km^2^ [36]. However, it is important to note that this relationship may not be linear nor direct. Indeed, study carried-out in malaria endemic countries across Africa suggested that population densities of 100 persons per km^2^ were more predictive of malaria infection in young children than very low densities (less than 10 persons per km^2^) or very high densities (more than 1000 persons per km^2^) [64]. Another study in Ethiopian highland suggests that, the spatial distribution of malaria in the low season is well-explained by both temperature and population density [65].

Persistence of malaria hotspots during low transmission periods might constitute a stepping-stone control strategies, and stir transmission during high transmission periods [22]. Therefore, these hotspots in low transmission seasons could be targeted for efficacious strategies, following a bottleneck approach to reduce malaria transmission at the local scale (see Additional file 3) [4, 66].

Our findings regarding the space-time dynamic of malaria, the three incidence periods of malaria and the lag time elapsing periods between the peak of rainfalls and the peak of malaria incidence might be considered for disrupting malaria transmission in the study area by adapting the malaria SMC program to local context and developing bottleneck strategies. Indeed, new strategies such as mass drug administration (MDA), mass screening and treatment (MSAT) are under consideration [23].

This study also found that malaria hotspots were constituted by all types of households whatever their socioeconomic status, this emphasizes that vector breeding sites were common in this area and the behaviour of the majority population influenced the profile and intensity of malaria transmission. Nevertheless, within this setting, low socioeconomic status of households and meteorological factors were positively correlated with malaria hotspots. After adjusting for meteorological components, the association of low socioeconomic status with malaria hotspots still remained. Poorer socioeconomic status of households was significantly associated with some factors that lead to increase and sustain malaria transmission, from poor-quality housing (bedroom with clay brick walls, roof made of clay/straw and wood, dirt floor, absence electricity, and absent of toilet, absence of tap) and absence of exposure to TV prevention campaigns. This positive association between malaria transmission and low socioeconomic status has been previously described in Burkina Faso at national or sub-national level [5, 17, 18, 34, 67].

The association with the rainfall/humidity disappeared in the multivariable analysis. Possible explanations of this observation could be inter alia, (1) the location of the households near water points, (2) permanent waterbodies due to the construction of the new dam of Soum which created flooded areas.

One limitation of the study was the fact that the study included both malaria cases diagnosed actively and passively. The passive detection of malaria cases might bias the findings by people who live closer to a health facility. However, this bias could be considered low because, in our context, about two-thirds of the study participant houses were located less than five kilometres. Moreover, active case detection, even in remote areas from health facilities, have also limited a potential bias due to health facility proximity. Additionally, as prevalence of home or self-treatment was presumably high, malaria incidence may be underestimate. A study carried out in 2011 noted that 72.7% of presumptive malaria admitted in a hospital of district practiced self-medication at home [68]. However, in our study area, strategies have been implemented to limit the practice of self-medication. Indeed, DSA’s field workers and community-based health workers, permanently sensitized population to avoid self-medication and attend a health facility if they experienced abnormal symptoms.

## Conclusion

Our study area was characterized by high incidence of malaria despite increasing efforts to fight the disease during the last decade. Findings showed a clear annual seasonal pattern of malaria incidence with three periods of different level of incidence and determined the lag times (9 weeks) between suitable meteorological factors and the peak of incidence of malaria. At a fine scale, and according to the three periods of malaria incidence, our findings supports a relative spatio-temporal stability of malaria hotspots, which were characterized by low socioeconomic status. Understanding environmental and socio-economic factors associated to the spatio-temporal dynamic of malaria is of high importance to adapt current control strategies and to develop new strategies such as bottleneck strategies.

## Additional files

Additional file 1: Autocorrelogram of weekly malaria incidence. (JPG 46 kb)

Additional file 2: First and second meteorological components derived from the Principal component analysis (PCA) of weekly meteorological variables. Rh max (Maximal relative humidity), Rh min (Minimal relative humidity), Rh ave. (Average relative humidity), Temp max (Maximal temperature), Temp min (Minimal temperature), Temp ave. (Average temperature), Rh max (Maximal relative humidity). (JPG 43 kb)

Additional file 3: Spatial hotspots according to the transmission periods (as described in Table 1) year from 2010 to 01-04 to 2014-12-31. Source: Burkina Faso, Base Nationale de Découpage du territoire (BNDT, 2006); shapefile downloaded from www.maplibrary.org. The map background (raster) is captured from https://www.openstreetmap.org/#map=12/12.6228/-2.1622. Maps created by Toussaint Rouamba, 2018. (PDF 931 kb)

## Abbreviations

AIC: Akaike Information Criterion
aOR: Adjusted Odds Ratio
CCF: Cross Correlation Function
CI: Confidence Interval
CISSE: Centre d’Information Sanitaire et de la Surveillance Epidémiologique
DGM: Direction Générale de la Météorologie
DSA: Demographic Surveillance Area
GEE: Generalized Estimating Equation
GPS: Global Positioning System coordinate
HAC: Hierarchical Ascendant Classification
HFP: Health Focal Points
MCA: Multiple Correspondence Analysis
NHD: Nanoro Health District
OR: Odds Ratio
PCA: Principal Component Analysis
PELT: Pruned Exact Linear Time
QIC: Quasi Akaike Information Criterion
RR: Relative Risk
SARIMA: Seasonal Autoregressive Integrated Moving Average
TLOH: Télégramme Lettre Official Hebdomendaire
WMO: World Meteorological Organization
