# Supplementary figures and images for: Socioeconomic and environmental factors associated with malaria hotspots in the Nanoro demographic surveillance area, Burkina Faso
Source: BMC Public Health. 2019 Feb 28;19:249. doi: 10.1186/s12889-019-6565-z (PMC6396465; doi:10.1186/s12889-019-6565-z)

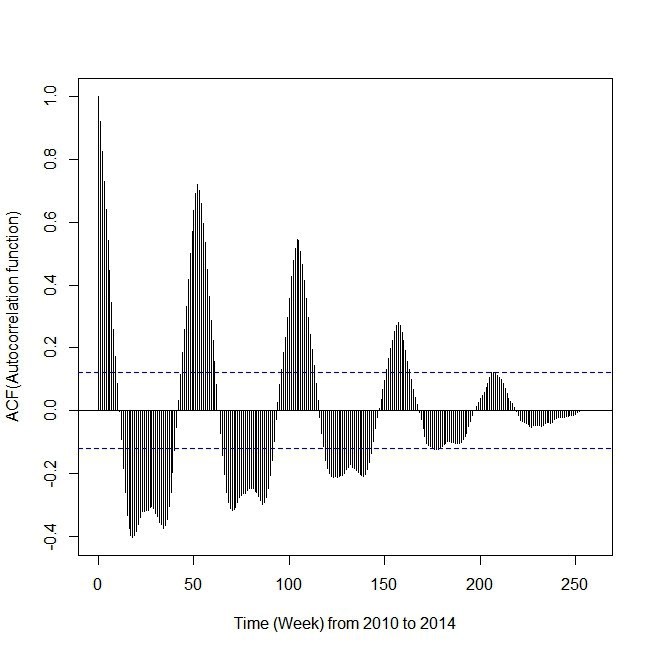

Supplement: Supplementary file 1 — Autocorrelogram of weekly malaria incidence. (JPG 46 kb) [file 12889_2019_6565_MOESM1_ESM.jpg]

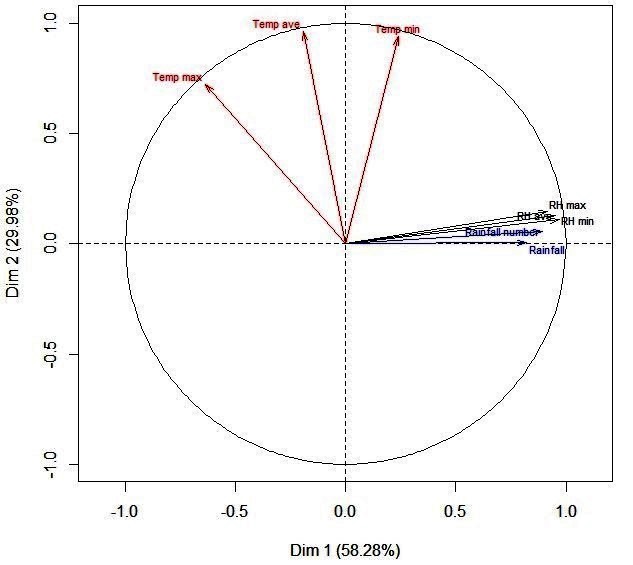

Supplement: Supplementary file 2 — First and second meteorological components derived from the Principal component analysis (PCA) of weekly meteorological variables. Rh max (Maximal relative humidity), Rh min (Minimal relative humidity), Rh ave. (Average relative humidity), Temp max (Maximal temperature), Temp min (Minimal temperature), Temp ave. (Average temperature), Rh max (Maximal relative humidity). (JPG 43 kb) [file 12889_2019_6565_MOESM2_ESM.jpg]
