# Supplementary material for: Socioeconomic and environmental factors associated with malaria hotspots in the Nanoro demographic surveillance area, Burkina Faso
Source: BMC Public Health. 2019 Feb 28;19:249. doi: 10.1186/s12889-019-6565-z (PMC6396465; doi:10.1186/s12889-019-6565-z)
Supplement: Supplementary file 3 — Spatial hotspots according to the transmission periods (as described in Table 1) year from 2010 to 01-04 to 2014-12-31. Source: Burkina Faso, Base Nationale de Découpage du territoire (BNDT, 2006); shapefile downloaded from www.maplibrary.org. The map background (raster) is captured from https://www.openstreetmap.org/#map=12/12.6228/-2.1622. Maps created by Toussaint Rouamba, 2018. (PDF 931 kb) [file 12889_2019_6565_MOESM3_ESM.pdf]

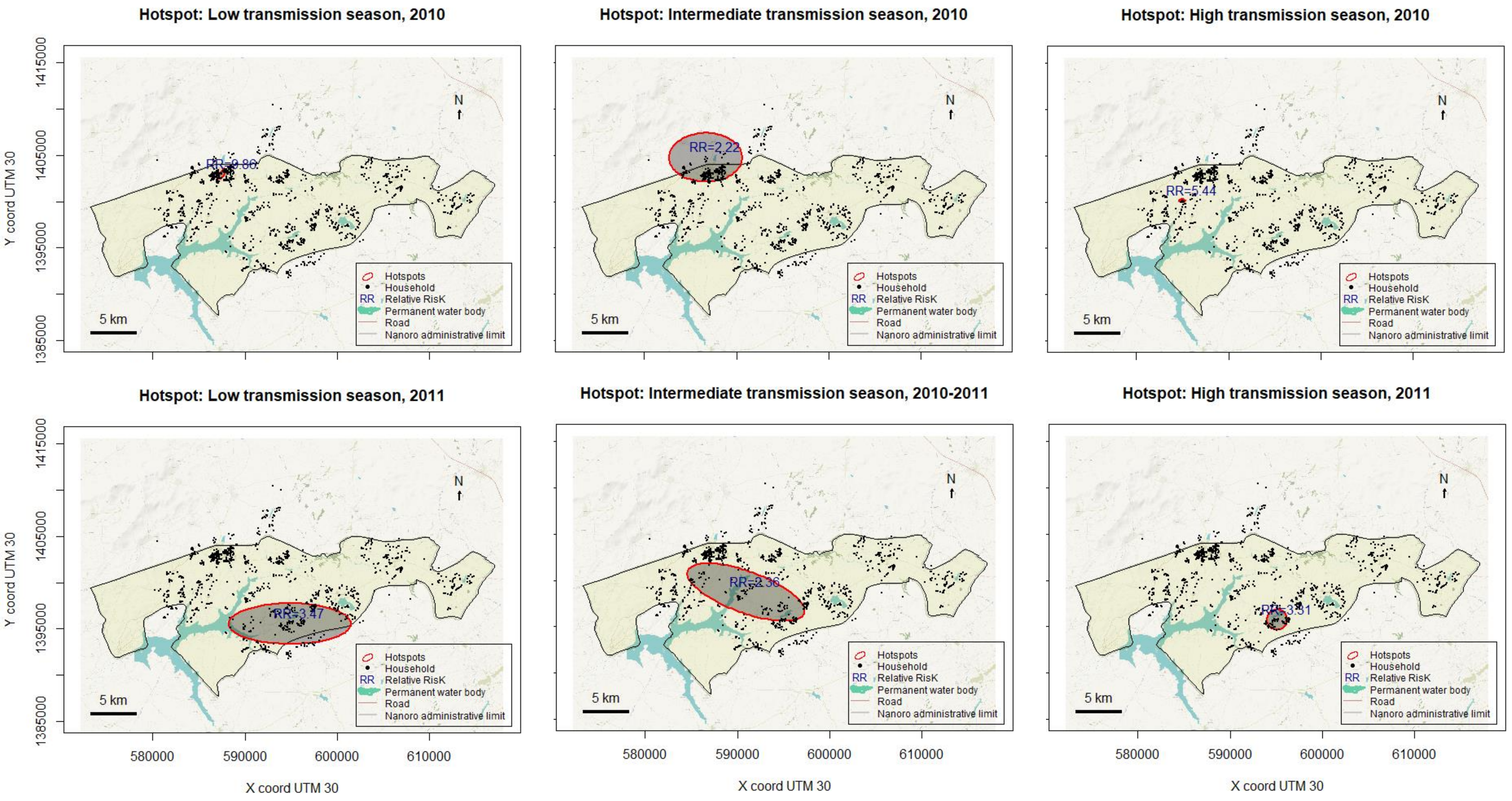
Hotspot: Low transmission season, 2010
RR=9.80
Hotspot: Intermediate transmission season, 2010
RR=2.22
Hotspot: High transmission season, 2010
RR=5.44
Hotspot: Low transmission season, 2011
RR=3.47
Hotspot: Intermediate transmission season, 2010-2011
RR=2.36
Hotspot: High transmission season, 2011
RR=3.31
N
5 km
Hotspots
Household
RR Relative RisK
Permanent water body
Road
Nanoro administrative limit
Y coord UTM 30
1385000
1395000
1405000
1415000
X coord UTM 30
580000
590000
600000
610000

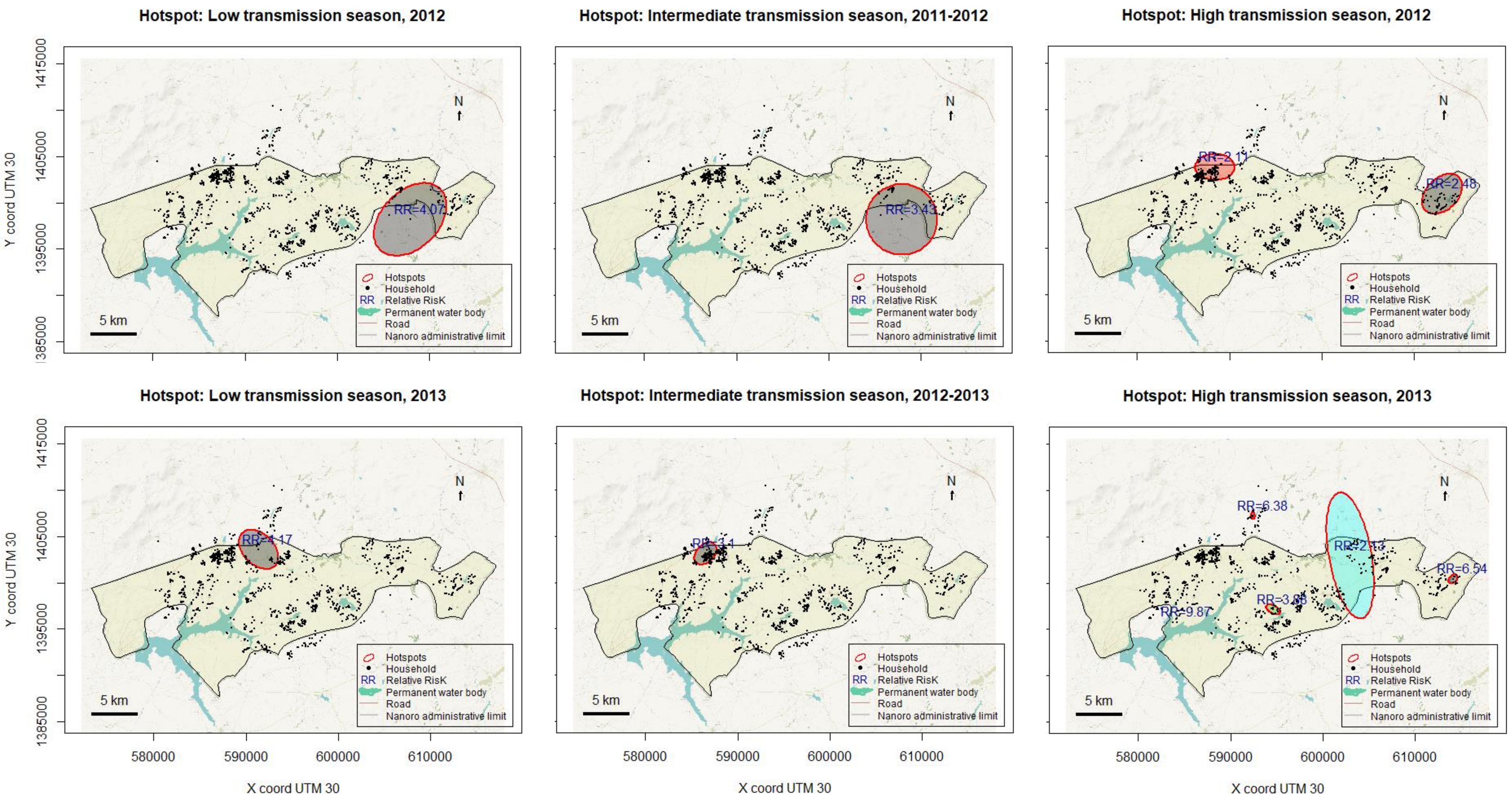
Hotspot: Low transmission season, 2012
Hotspot: Intermediate transmission season, 2011-2012
Hotspot: High transmission season, 2012
Hotspot: Low transmission season, 2013
Hotspot: Intermediate transmission season, 2012-2013
Hotspot: High transmission season, 2013
RR=4.07
RR=3.43
RR=2.11
RR=2.48
RR=4.17
RR=3.1
RR=6.38
RR=2.13
RR=6.54
RR=3.88
RR=9.87
N
5 km
Hotspots
Household
RR Relative RisK
Permanent water body
Road
Nanoro administrative limit
Y coord UTM 30
X coord UTM 30
1385000
1395000
1405000
1415000
580000
590000
600000
610000

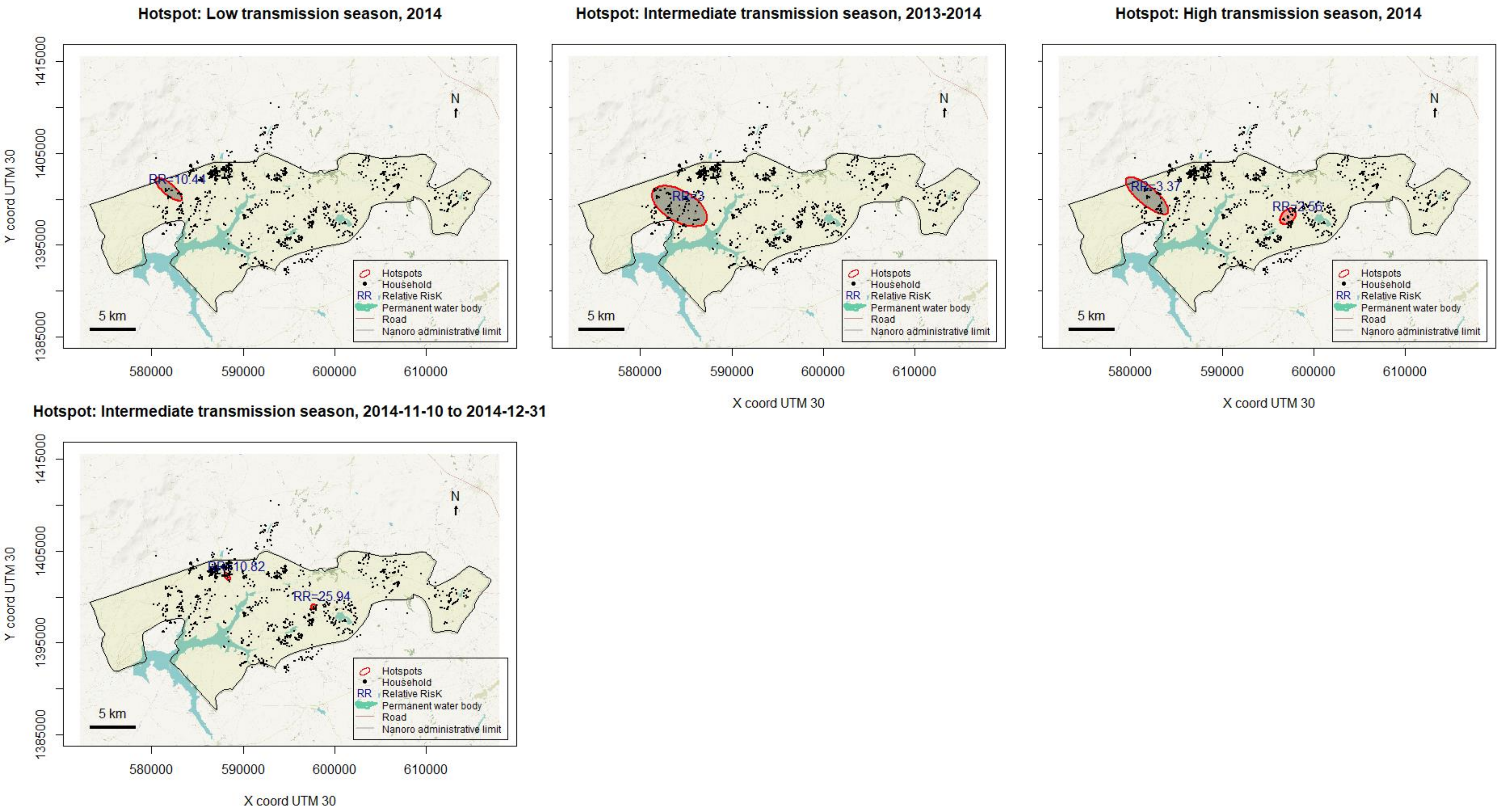
Hotspot: Low transmission season, 2014
Hotspot: Intermediate transmission season, 2013-2014
Hotspot: High transmission season, 2014
Hotspot: Intermediate transmission season, 2014-11-10 to 2014-12-31
RR=10.44
RR=3
RR=3.37
RR=2.56
RR=10.82
RR=25.94
N
5 km
Hotspots
Household
RR Relative RisK
Permanent water body
Road
Nanoro administrative limit
Y coord UTM 30
X coord UTM 30
1385000
1395000
1405000
1415000
580000
590000
600000
610000
